# Novel mutual prodrug of 5-fluorouracil and heme oxygenase-1 inhibitor (5-FU/HO-1 hybrid): design and preliminary *in vitro* evaluation

**DOI:** 10.1080/14756366.2021.1928111

**Published:** 2021-06-24

**Authors:** Loredana Salerno, Luca Vanella, Valeria Sorrenti, Valeria Consoli, Valeria Ciaffaglione, Antonino N. Fallica, Vittorio Canale, Paweł Zajdel, Rosario Pignatello, Sebastiano Intagliata

**Affiliations:** a Department of Drug and Health Sciences, University of Catania, Catania, Italy; b Department of Organic Chemistry, Jagiellonian University Medical College, Kraków, Poland

**Keywords:** Mutual prodrugs, hybrid compounds, anticancer agents, 5-fluorouracil, heme oxygenase 1, HO-1 inhibitors

## Abstract

In this work, the first mutual prodrug of 5-fluorouracil and heme oxygenase1 inhibitor (5-FU/HO-1 hybrid) has been designed, synthesised, and evaluated for its *in vitro* chemical and enzymatic hydrolysis stability. Predicted *in silico* physicochemical properties of the newly synthesised hybrid (**3**) demonstrated a drug-like profile with suitable Absorption, Distribution, Metabolism, and Excretion (ADME) properties and low toxic liabilities. Preliminary cytotoxicity evaluation towards human prostate (DU145) and lung (A549) cancer cell lines demonstrated that **3** exerted a similar effect on cell viability to that produced by the reference drug 5-FU. Among the two tested cancer cell lines, the A549 cells were more susceptible for **3**. Of note, hybrid **3** also had a significantly lower cytotoxic effect on healthy human lung epithelial cells (BEAS-2B) than 5-FU. Altogether our results served as an initial proof-of-concept to develop 5-FU/HO-1 mutual prodrugs as potential novel anticancer agents.

## Introduction

5-Fluorouracil (5-FU) is a chemotherapy medication belonging to pyrimidine antimetabolites, a well-known class of anticancer drugs acting on enzymes involved in DNA synthesis and metabolism^1^^,^^2^. Since its discovery in the 1960s, 5-FU has been effectively used to treat a wide variety of malignancies, including gastric, pancreatic, breast, and colorectal adenocarcinoma^3^. Moreover, the continuous infusion of 5-FU has been recently suggested as a novel treatment for heavily pre-treated prostate cancer patients^4^. Despite the clinical significance of 5-FU treatments, its use is often limited by unfavourable pharmacokinetic profile and high non-specific toxicity^5^. Conversely, in some cases, the 5-FU therapeutic index can be improved by prolonged infusion administration^6^; however, the risk of severe toxicity for a life-threatening regime cannot be neglected^7^.

Alternatively, drug combination therapy is often considered an efficient approach to increasing drugs’ clinical efficacy through additive or synergistic effects^8^^,^^9^. For instance, identified *in vitro* synergistic antitumor effect of a combination of 5-FU and cisplatin against non-small cell lung cancer cell line (A549) has been proposed as a possible strategy for overcoming 5-FU resistance in cancer therapy^10^. On the other hand, drug coadministration can be associated with low patient compliance and highly drug–drug interaction risks^11^^,^^12^. Thus, the development of multitarget ligands that simultaneously act at different biological targets has gained momentum and might represent an innovative strategy to overcome specific drawbacks associated with the coadministration of two or more agents^13^.

**Figure 1.**
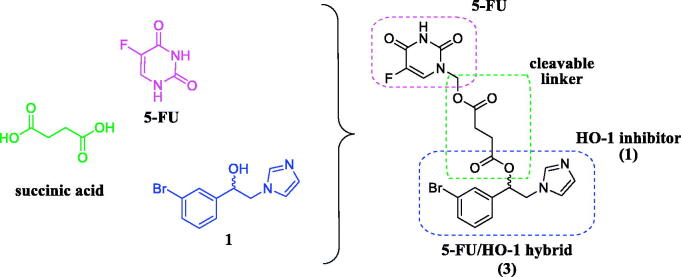
Chemical structure of 5-FU, succinic acid, HO-1 inhibitor (**1**), and 5-FU/HO-1 hybrid (**3**).

Heme oxygenases (HOs) are heat-shock proteins (Hsps) with catalytic activity, mainly involved in the catabolism of heme into ferrous iron (Fe^2+^), carbon monoxide (CO), and biliverdin (BV), this last rapidly converted into bilirubin (BR) by biliverdin reductase (BVR)^14^. Among the three different isoforms known to date (i.e. HO-1, HO-2, and HO-3, respectively), only the first two possess the enzymatic activity and a clinical significance^15^^,^^16^. Specifically, HO-1 is an inducible isoform of the enzyme, predominantly expressed in the liver and spleen, while low levels are detected in many organs and tissues under physiological conditions^17^. In consequence of specific stimuli, such as oxidative stress, ultraviolet radiations, heavy metals, and xenobiotics, the HO-1 level increases^17^. An abnormal HO-1 level has been linked to cancer formation and maintenance due to the perturbation of cellular homoeostasis, which affects the balance between apoptosis and cell proliferation^18^. Therefore, the pharmacological inhibition of HO-1 is emerging as an attractive strategy for cancer chemotherapy^19^^,^^20^. Indeed, HO-1 inhibitors showed antiproliferative properties on different cancer cell lines^21–23^ and produced additive or synergistic effects in association with anticancer agents, such as in the case of a combination of **1** (Figure 1) and doxorubicin^23^. Moreover, multitarget ligands based on HO-1 inhibitors efficiently overcame imatinib-resistance in chronic myeloid leukaemia (CML) cancer cells^24^.

Herein, we reported the synthesis, *in vitro* stability studies, and preliminary biological evaluation of the first 5-FU/HO-1 hybrid (**3**, Figure 1), which served as an initial proof-of-concept to develop novel polypharmacological agents to improve existing cancer chemotherapies^25^. Specifically, according to the mutual prodrugs approach^26^^,^^27^, we conjugated two different pharmacologically active compounds, such as the anticancer agent 5-FU and the azole-based HO-1 inhibitor (**1**), utilising succinic acid as a biocompatible and biodegradable linker (Figure 1).

## Materials and methods

### Chemistry

Reagents, solvents, and starting materials were purchased from commercial suppliers. Melting points were determined in an IA9200 Electrothermal apparatus equipped with a digital thermometer in capillary glass tubes and are uncorrected. Infrared spectra were recorded on a Perkin Elmer 281 Fourier-transform infrared (FTIR) spectrometer in KBr discs (KBr, selected lines) or placing a sample droplet between two discs of pure NaCl (neat sample). Elemental analyses for C, H, N, were within ± 0.4% of theoretical values and were performed on a Carlo Erba Elemental Analyser Mod. 1108 apparatus. ^1^H NMR spectra were recorded on Varian Inova Unity (200 MHz) spectrometers in dimethyl sulfoxide-d_6_ (DMSO-*d_6_*) or methanol-*d_4_* (CD_3_OD) solution. Chemical shifts are in δ values (ppm) using tetramethylsilane (TMS) or CH_3_OH as the internal standard for spectra recorded in DMSO-*d_6_* or CD_3_OD, respectively. Coupling constants (*J*) are given in Hz. Signal multiplicities are characterised as follows: s (singlet), d (doublet), t (triplet), q (quartet), m (multiplet), br (broad). Reactions were monitored by thin-layer chromatography (TLC), carried out on Merck plates (Kieselgel 60 F254), using UV light (λ = 254 and 366 nm) for visualisation and staining the TLC plate with iodine vapour in a closed chamber. Flash column chromatography was performed on silica gel 60, 0.040–0.063 mm (Merck, Kenilworth, NJ, USA). Mass spectra were recorded on a UPLC-MS/MS system consisted of a Waters ACQUITY® UPLC^®^ (Waters Corporation, Milford, MA, USA) coupled to a Waters TQD mass spectrometer (electrospray ionisation mode ESI-tandem quadrupole). Chromatographic separations were carried out using the Acquity UPLC BEH (bridged ethyl hybrid) C18 column; 2.1 × 100 mm, and 1.7 mm particle size, equipped with Acquity UPLC BEH C18 Van Guard pre-column, 2.1 × 5 mm, and 1.7 mm particle size. The column was maintained at 40 °C and eluted under gradient conditions from 95% to 0% of eluent A over 10 min, at a flow rate of 0.3 ml min^−1^. Eluent A: water/formic acid (0.1%, v/v); eluent B: acetonitrile/formic acid (0.1%, v/v). Chromatograms were made using Waters eλ PDA detector. Spectra were analysed in 200–700 nm range with 1.2 nm resolution and sampling rate 20 points/s. MS detection settings of Waters TQD mass spectrometer were as follows: source temperature 150 °C, desolvation temperature 350 °C, desolvation gas flowrate 600 L h^−1^, cone gas flow 100 L h^−1^, capillary potential 3.00 kV, cone potential 40 V. Nitrogen was used for both nebulising and drying gas. The data were obtained in a scan mode ranging from 50 to 2000 *m*/*z* in time 1.0 s intervals. Data acquisition software was MassLynx V 4.1 (Waters). The UPLC/MS purity of all the final compounds was confirmed to be 95% or higher.

### Synthesis of 4‐[1‐(3‐bromophenyl)‐2‐(1H‐imidazol‐1‐yl)ethoxy]‐4‐oxobutanoic acid (2)

A mixture of compound **1** (0.19 g, 0.70 mmol), succinic anhydride (0.07 g, 0.70 mmol), and triethylamine (0.116 ml, 0.84 mmol) was refluxed in dry methylene chloride (10 ml) for 6 h. The solvent was removed under reduced pressure, and the crude thus obtained was purified by column chromatography on silica gel using a mixture of ethyl acetate-methanol (7:3, v/v) as eluent to afford compound **2** (0.39 g, 33%), as a white pure solid: mp 64.0–66.5 °C. IR (neat, selected lines) cm^−1^ 3126, 2930, 2738, 1738, 1574, 1428, 1371, 1155, 836. ^1^H NMR (200 MHz, CD_3_OD) δ 7.62 (*s*, 1H, imidazole), 7.55–7.41 (*m*, 2H, aromatic), 7.30–7.20 (*m*, 2H, aromatic), 7.13 (*s*, 1H, imidazole), 6.97 (*s*, 1H, imidazole), 6.03 (*t*, *J* = 5.4 Hz, 1H, *CH*OHCH_2_), 4.45 (d, *J* = 5.5 Hz, 2H, CHOH*CH_2_*), 2.72–2.45 (*m*, 2H + 2H, CO*CH_2_CH_2_*CO). UPLC/MS purity 98%, *t*_R_ = 3.543 min. MS (ESI) *m*/*z*: 367.2 [M + H]^+^. Anal. Calcd. for C_15_H_15_BrN_2_O_2_: C, 49.06; H, 4.12; N, 7.63. Found: C, 48.99; H, 4.09; N, 7.60.

### Synthesis of 1‐[1‐(3‐bromophenyl)‐2‐(1H‐imidazol‐1‐yl)ethyl]4‐(5‐fluoro‐2,4‐dioxo‐1,2,3,4‐tetrahydropyrimidin‐1‐yl)methyl butanedioate (3)

To a stirred suspension of 1-(hydroxymethyl)-5-FU (0.09 g, 0.56 mmol) in a mixture solvent of dry methylene chloride-acetonitrile (2 + 2 ml), compound **2** (0.25 g, 0.67 mmol), *N*-(3-dimethylaminopropyl)-*N′*-ethylcarbodiimide hydrochloride (EDC·HCl) (0.13 g, 0.67 mmol), and a catalytic amount of 4-dimethylaminopryridine (DMAP) (0.005 g, 0.04 mmol), were added under a nitrogen flow, and the mixture was then stirred at room temperature for 12 h. The solvent was removed under reduced pressure, and the crude thus obtained was purified by column chromatography on silica gel using a mixture of ethyl acetate-methanol (9:1, v/v) as eluent to afford compound **3** (0.06 g, 22%), as a white pure solid: mp 172.0–174.5° C. IR (KBr, selected lines) cm^−1^ 3448, 3122, 1732, 1671, 1509, 1412, 1366, 1265, 1143, 993. ^1^H NMR (200 MHz, DMSO-*d6*): *δ* 8.11 (d, *J_H-F_* = 6.6 Hz, 1H, *CH*CF), 7.62 − 7.44 (*m*, 1H + 2H, imidazole + aromatic), 7.40 − 7.25 (*m*, 2H, aromatic), 7.12 (*s*, 1H, imidazole), 6.85 (*s*, 1H, imidazole), 5.95 (*t*, *J* = 5.7 Hz, 1H, *CH*OHCH_2_), 5.56 (*s*, 2H, C*H_2_*O), 4.37 (d, *J* = 5.8 Hz, 2H, CHOH*CH_2_*), 2.71–2.55 (*m*, 2H + 2H, COC*H*_2_C*H*_2_CO). UPLC/MS purity 99%, *t*_R_ = 3.773 min. MS (ESI) *m*/*z*: 509.0 [M + H]^+^. Anal. Calcd. for C_20_H_18_BrFN_4_O_6_: C, 47.17; H, 3.56; N, 11.00. Found: C, 47.03; H, 3.49; N, 10.95.

### HPLC method

The HPLC analysis of samples at various time intervals from *in vitro* stability in different buffers and porcine esterase solution was performed on Shimadzu Prominence-i LC-2030C 3 D Plus equipped with RID20A. Detector chromatographic separation were carried out using Chromolith SpeedROD RP 18.5 µm, 1.6 × 50 mm, Merck. Spectra were analysed in 200–800 nm range with 1.2 nm resolution. The column was maintained at 30 °C and eluted under gradient conditions from 100% to 0% of eluent A over 3 min, at a flow rate of 5 ml min^−1^. Eluent A: water/trifluoroacetic acid (0.1%, v/v); eluent B: acetonitrile/ trifluoroacetic acid (0.1%, v/v).

### Chemical stability assessment of 3

A stock solution of compound **3** in DMSO (3.0 mg/mL) was prepared. To a test tube containing 0.9 ml of the corresponding Acetate (pH = 2.0) or PBS buffer solution (pH = 7.4 and 8.0, respectively), 0.1 ml of stock solution was added, and the mixture stirred and thermostated in a sand bath at 37 °C. Aliquots (0.1 ml) were withdrawn at specific time intervals and transferred to sample vials containing acetonitrile (0.9 ml). The percentage of compound remaining was followed by HPLC analysis. The retention time (*t*_R_) of compound **1**, **2**, and **3** were 0.74, 0.89, and 0.95 min, respectively. All the experiments were performed in triplicate.

### In vitro stability of 3 in porcine esterase solution

0.001 g of lyophilised powder of esterase from the porcine liver (Sigma-Aldrich, St. Louis, Missouri, USA) was reconstituted in 1.0 ml PBS buffer (0.01 M, pH 7.4) to make an aqueous porcine esterase solution (5 U/mL), and then pre-thermostated at 37 °C. To a test tube containing 1.85 ml of PBS buffer, 0.15 ml from the stock solution of the test compound was added. The mixture was then stirred and thermostated in a sand bath at 37 °C, and 1.0 ml of the porcine esterase solution was added to initiate the enzymatic reaction. For the negative control reaction, the volume of porcine esterase solution was replaced by phosphate buffer. Aliquots (0.3 ml) were withdrawn at 0, 30, 60, 120, 180, and 240 min, and quenched with cold acetonitrile (0.7 ml)^28^. The samples were centrifuged for 6 min at 10,000 rpm, and supernatants were analysed by HPLC to check the amounts (area under the curve, AUC) of the remaining intact compound. All the experiments were performed in triplicate. Pseudo-first-order rate constant for the hydrolysis was determined from the slope of linear plots of the natural logarithm (ln) of the AUC of the peak at time t (AUC_t_) against time. Half-life (t_1/2_) was calculated according to Equation (1):

(1) $$t_{1/2}\;=\;\;\text{ln }2/k$$

were *k* is the pseudo-first-order rate constant.

### Biological evaluation

#### Preparation of spleen microsomal fractions

Since the dominance of HO-1 protein in the rat spleen has been well documented^29–32^, HO-1 was obtained from rat spleen as the microsomal fraction prepared by differential centrifugation. This particular microsomal preparation was selected in order to use the most native (i.e. closest to *in vivo*) forms of HO-1. Spleen (Sprague-Dawley rats) microsomal fractions were prepared according to the procedure outlined by Ryter *et al.*^33^. The experiments reported in the present paper complied with current Italian law and met the guidelines of the Institutional Animal Care and Use Committee of MINISTRY OF HEALTH (Directorate General for Animal Health and Veterinary Medicines) (Italy). The experiments were performed in male Sprague-Dawley albino rats (150 g body weight and age 45 d). They had free access to water and were kept at room temperature with a natural photo-period (12-h light/12-h dark cycle). For measuring HO-1 activity, each rat was sacrificed and their spleen were excised and weighed. A homogenate (15%, w/v) of spleens pooled from four rats was prepared in ice-cold HO-homogenising buffer (50 mM Tris buffer, pH 7.4, containing 0.25 M sucrose) using a Potter-Elvehjem homogenising system with a Teflon pestle. The microsomal fraction of rat spleen homogenate was obtained by centrifugation at 10,000 *g* for 20 min at 4 °C, followed by centrifugation of the supernatant at 100,000 *g* for 60 min at 4 °C. The 100,000 *g* pellet (microsomes) was resuspended in 100 mM potassium phosphate buffer, pH 7.8, containing 2 mM MgCl_2_ with a Potter-Elvehjem homogenising system. The rat spleen microsomal fractions were divided into equal aliquots, placed into microcentrifuge tubes, and stored at −80 °C for up to 2 months.

#### Preparation of BVR

Liver cytosol has been used as a source of BVR. Rat liver was perfused through the hepatic portal vein with cold 0.9% NaCl, then it was cut and flushed with 2 × 20 ml of ice-cold PBS to remove all of the blood. Liver tissue was homogenised in 3 volumes of a solution containing 1.15% KCl w/v and Tris buffer 20 mM, pH 7.8 on ice. Homogenates were centrifuged at 10,000 *g*, for 20 min at 4 °C. The supernatant was decanted and centrifuged at 100,000 *g* for 1 h at 4 °C to sediment the microsomes. The 100,000 g supernatant was saved and then stored in small amounts at −80 °C after its protein concentration was measured.

#### Measurement of HO-1 enzymatic activities in the microsomal fraction of rat spleen

The HO-1 activity was determined by measuring the bilirubin formation using the difference in absorbance at 464 to 530 nm as described by Ryter et al.^33^. Reaction mixtures (500 µL) consisted of 20 mM Tris-HCl, pH 7.4, (1 mg/mL) microsomal extract, 0.5–2.0 mg/mL biliverdin reductase, 1 mM NADPH, 2 mM glucose 6-phosphate (G6P), 1 U G6P dehydrogenase, 25 µM haemin, 10 µL of DMSO (or the same volume of DMSO solution of test compounds to a final concentration of 100, 10, and 1 µM). Incubations were carried out for 60 min at 37 °C in a circulating water bath in the dark. Reactions were stopped by adding 1 volume of chloroform. After recovering the chloroform phase, the amount of bilirubin formed was measured with a double-beam spectrophotometer as OD464-530 nm (extinction coefficient, 40 mM/cm^−1^ for bilirubin). One unit of the enzyme was defined as the amount of enzyme catalysing the formation of 1 nmol of bilirubin/mg protein/h.

#### Cell cultures and cell viability assay

Experiments were performed on human prostate cancer cells (DU145; ATCC HTB-81), human lung cancer cells (A549; ATCC CCL-185-LUC2) and human bronchial epithelium cells (BEAS-2B; ATCC CRL-9609). Cells were grown in Dulbecco's modified Eagle's medium (DMEM) supplemented with 10% of heat-inactivated foetal bovine serum (FBS), 100 U/ml penicillin and 100 μg/ml streptomycin (Sigma-Aldrich, Steinheim, Germany). Cells were incubated at 37 °C in a humidified atmosphere with 5% CO_2._ The effect of 5-FU, compound **1** and **3** on cell viability was assessed by performing the 3–(4,5-dimethylthiazole-2-yl)-2,5-diphenyltetrazolium bromide (MTT) assay. Cells were seeded into 96-well plates at a density of 7,0 × 10^3^ cells/well in 100 µl of culture medium. After 24 h, cells were treated with the compounds at three different concentrations (1 µM, 10 µM and 50 µM) for 72 h. Following treatments, 0.5 mg/ml of 3-[4,5-dimethylthiazol-2-yl]-2,5-diphenyltetrazolium bromide (MTT) (Sigma Aldrich) was added to each well and incubated for 4 h at 37 °C. Finally, DMSO was used to dissolve formazan salts and absorbance was measured at 570 nm in a microplate reader (Biotek Synergy-HT). Eight replicate wells were used for each group. Four independent experiments were performed.

#### Statistical analysis

Data are represented as mean ± standard error (SEM). One-way analysis of variance (ANOVA) was used to compare differences among groups, and statistical significance was assessed by the Tukey–Kramer *post hoc* test. The level of significance for all statistical tests was set at *p* ≤ 0.05.

## Results and discussion

### Chemistry

**Scheme 1.**
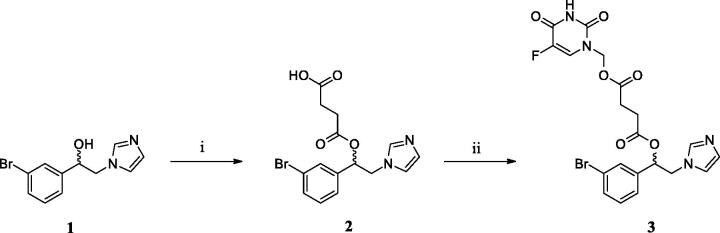
Reagents and conditions: (i) succinic anhydride, triethylamine, dry methylene chloride, reflux, 6 h; (ii) 1-hydroxymethyl-5-fluorouracil, EDC·HCl, DMAP, dry methylene chloride/acetonitrile (1:1, v/v), rt, 12 h.

1–(3-bromophenyl)-2-(1*H*-imidazol-1-yl)ethanol (**1**) was prepared according to previously reported synthetic procedures^23^^,^^34^. Subsequently, the final 5-FU/HO-1 hybrid (**3**) has been synthesised through a two-step pathway by following the reaction conditions depicted in Scheme 1. The imidazole-based derivative **1** reacted with succinic anhydride under basic condition to give 4–(1-(3-bromophenyl)-2-(1*H*-imidazol-1-yl)ethoxy)-4-oxobutanoic acid (**2**). Intermediate **2** was then coupled with 1-hydroxymethyl-5-fluorouracil, which was prepared based on a known method^35^, and using EDC·HCl as a carboxylic acid activator and DMAP as a catalyst^36^.

### In silico prediction of physicochemical, ADME, and toxicity properties

The pharmacokinetic profile and adverse side effects (ADME/Tox) of a molecule are closely related to its physicochemical properties; thus, calculation of molecular descriptors appears to be a useful methodology to define drug-likeness^37^. The predicted physicochemical properties for parent compounds (5-FU and **1**) and the 5-FU/HO-1 hybrid (**3**) were calculated and reported in Table 1.

**Table 1.** Predicted physicochemical properties of 5-FU, **1**, and **3**.

Compd.	Lipinski's rule^a^				Veber's rule^a^		MDDR-like rule^b^
	MW	cLogP	HBD	HBA	RBN	TPSA	
**5-FU**	130.08	−0.66	2	4	0	58.20	nondrug-like
**1**	267.12	1.94	1	3	3	38.05	mid-structure
**3**	508.28	1.86	1	9	11	119.83	drug-like
**Optimal**	≤500	≤5	≤5	≤10	≤3	≤140	–

^a^Molecular weight (MW), calculated LogP (cLogP), number of hydrogen bond donors (HBD), number of hydrogen bond acceptors (HBA), rotatable bonds number (RBN), topological polar surface area (TPSA). Calculator plugins were used for structure-property prediction and calculation, Marvin 20.21.0, ChemAxon (https://www.chemaxon.com).

^b^MDL Drug Data Report (MDDR) was predicted using PreADMET web-based application (http://preadmet.bmdrc.kr).

Both Lipinski’s and Veber’s rules were taken into account to predict the drug-likeness and the oral bioavailability of title compounds^38^^,^^39^. Analysis of physicochemical descriptors revealed that for the 5-FU/HO-1 hybrid (**3**), only one violation of the Lipinski’s rule of five occurred (i.e. MW > 500), while both the 5-FU and the imidazole-based derivative (**1**) fully comply with the rule. Similarly, one violation of Veber’s rule was observed for compound **3** (i.e. RBN >3). Notably, unlike specific descriptors, including the cLogP, HBD, and HBA, a statistically significant increase in MW and RBN values have been observed for approved oral drugs in this decade, as recently analysed by Shultz^40^. Indeed, several examples of orally-administered marketed drugs showed one or two violations of the rule of five, including prodrugs such as dabigatran etexilate, fosinopril, and olmesartan medoxomil^41^. Consistently, a suitable drug-like profile for **3** (Table 1) has been found according to the MDDR like rule^42^^,^^43^.

Poor pharmacokinetics or high toxicity of drugs are two of the primary cause of clinical development failure; thus, an early *in silico* assessment of ADME and toxicity properties (PreADMET) of 5-FU, **1**, and **3** was performed. Results suggested that the newly synthesised hybrid (**3**) might exhibit a proper ADME profile with good absorption and sufficient distribution (Table 2). Also, unlike 5-FU, no toxic liabilities were predicted for the novel hybrid (Table 3), suggesting that the mutual prodrug strategy can effectively mitigate 5-FU high non-specific toxic-effects.

**Table 2.** *In silico* ADME prediction for 5-FU, **1**, and **3**.

	Absorption^a^		Distribution^a^	
Compd.	HIA (%)	P_app_ (nm/s)	PPB (%)	BBB (C_brain_/C_blood_)
**5-FU**	75.9	17.3	8.3	0.2
**1**^b^	96.1	29.6	65	0.7
**3**	97.7	20.6	83	0.1
**Range ** ** (meaning)**	70–100 % (well-absorbed)	4–70 (middle permeability)	>90 (strong binding)	2.0–0.1 (permeability to CNS)

^a^Human intestinal absorption (HIA), *in vitro* Caco-2 cell permeability (P_app_), *in vitro* plasma protein binding (PPB), *in vivo* blood-brain barrier penetration (BBB). Selected ADME properties were predicted using PreADMET web-based application (http://preadmet.bmdrc.kr).

^b^Data from reference ^34^.

**Table 3.** *In silico* toxicity prediction for 5-FU, **1**, and **3**.

Compd.	Toxicity prediction^a^			
	Mutagenic	Tumorigenic	Irritant	Reproductive Effects
**5-FU**	high	high	high	high
**1**^b^	none	none	none	none
**3**	none	none	none	none

^a^Properties were predicted using DataWarrior software^49^.

^b^Data from reference^34^.

### Chemical stability and in vitro enzymatic hydrolysis

**Figure 2.**
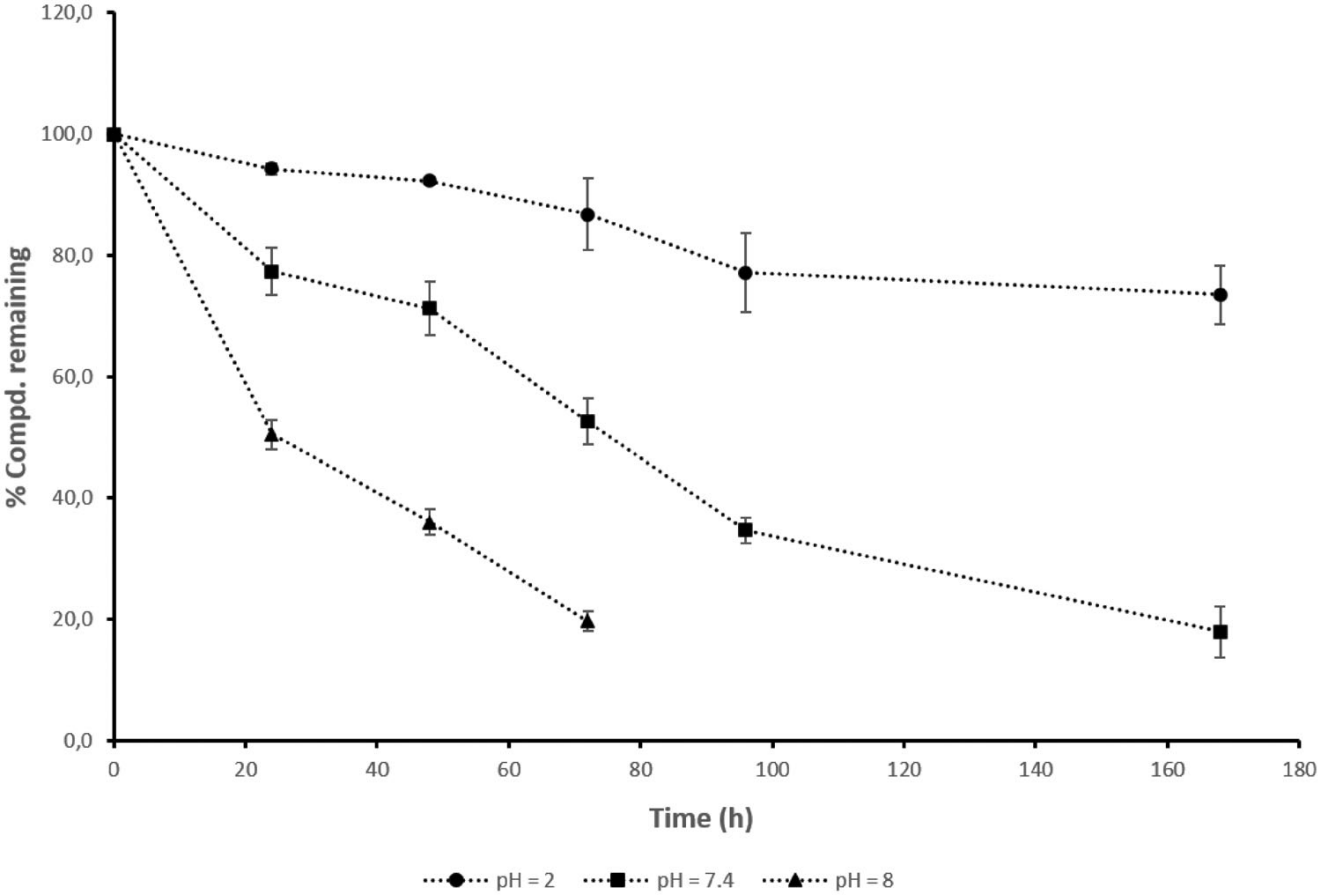
*In vitro* chemical stability of 5-FU/HO-1 hybrid (**3**) at different pHs. Data are representative of three independent experiments and values are expressed in mean ± SEM.

The chemical stability of **3** was evaluated at different pH values to mimic physiological conditions, including gastrointestinal (GI) tract (pH = 2.0), human plasma (pH = 7.4), and pancreatic fluid (pH = 8.0). As a result, the pH value strongly affected the chemical stability and hydrolysis rate of the hybrid **3** (Figure 2).

The lowest *in vitro* hydrolysis rate for **3** was observed at acid pH, suggesting the hybrid's stability in the gastric environment, thus eventually compatible with its oral administration. On the contrary, in alkaline conditions (i.e. pH = 8.0), the chemical stability of **3** was significantly lower than both in acid and neutral conditions, supporting the fact that a small percentage of free parent compounds might be available for absorption across the intestine. Indeed, the percentage of compound **3** remaining after 24 h equalled 94.2% at pH = 2.0, 77.3% at pH = 7.4, and 50.4% at pH = 8.0, respectively (Figure 2, Table S1 Supplemental material).

**Figure 3.**
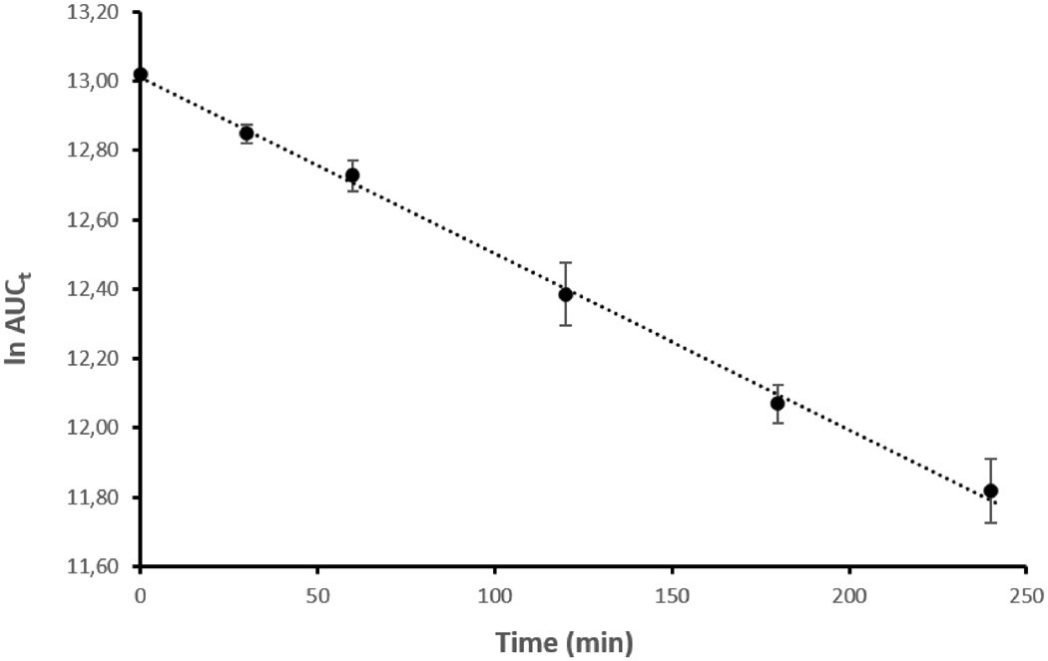
Hydrolysis rate of **3** in porcine esterase solution. A linear pseudo-first-order plot of the ln AUC_t_ *vs.* time was observed. k = 5.07 × 10^−3 ^min^−1^; t_1/2_ = 136 min; r = 0.999. Data are representative of three independent experiments and values are expressed in mean ± SEM.

To exert its pharmacologic effect the 5-FU moiety must be released from **3** and subsequently converted into different active metabolites (i.e. fluorodeoxyuridine monophosphate, fluorodeoxyuridine triphosphate, and fluorouridine triphosphate)^1^. Similarly, the parent compound **1** should be efficiently regenerated. Therefore, the enzymatic stability of **3** in porcine esterase solution was investigated. The hydrolysis rate profile observed for the 5-FU/HO-1 hybrid in such conditions was in line with the pseudo-first-order kinetics model (Figure 3). Remarkably, the rate of hydrolysis of **3** in plasma mimicking solution was quicker (t_1/2_ = 136 min) than that in buffer solution (t_1/2_ = 1,689 min, Figure S8 Supplemental material), confirming the enzyme hydrolysis contribution. These data support the choice of the succinyl spacer as a suitable cleavable linker to release the active moieties from **3** in the right time frame compatible with the biological activity. Notably, hybrid **3** was not detected at 24 h incubation in porcine esterase solution (Figure S12 Supplemental material).

### HO-1 inhibition activity

Inhibition activity assay for HO-1 was performed by extracting the enzyme from the rat spleen microsomal fraction. HO-1 activity was determined by measuring the formation of BR using the difference in absorbance at 464–530 nm, according to the protocol described in the experimental section. Results are expressed as enzyme inhibition activity (IC_50_) in μM (Table S2, Supplemental material). As expected, the hydride **3** exhibited a lower inhibitory potency towards HO-1 than the parent compound **1** (82 ± 2.1 μM *vs.* 0.4 ± 0.01 μM, respectively). Compound **2**, a possible metabolite of **3** showed even lower inhibitory activity towards HO-1 (104.6 ± 5.8 μM). These results were consistent with previous structure-activity relationship (SAR) studies performed on azole-based analogs ^44^^,^^45^, stressing that changes at the ethanolic chain are detrimental to the HO-1 inhibitory activity. Although compound **3** displayed a lower inhibitory potency towards the HO-1 with respect to parent derivative **1**, this aspect does not represent an issue since hybrid **3** acts as a mutual prodrug by releasing the parent drugs (i.e. 5-FU and **1**, respectively).

### Effects on cell viability

**Figure 4.**
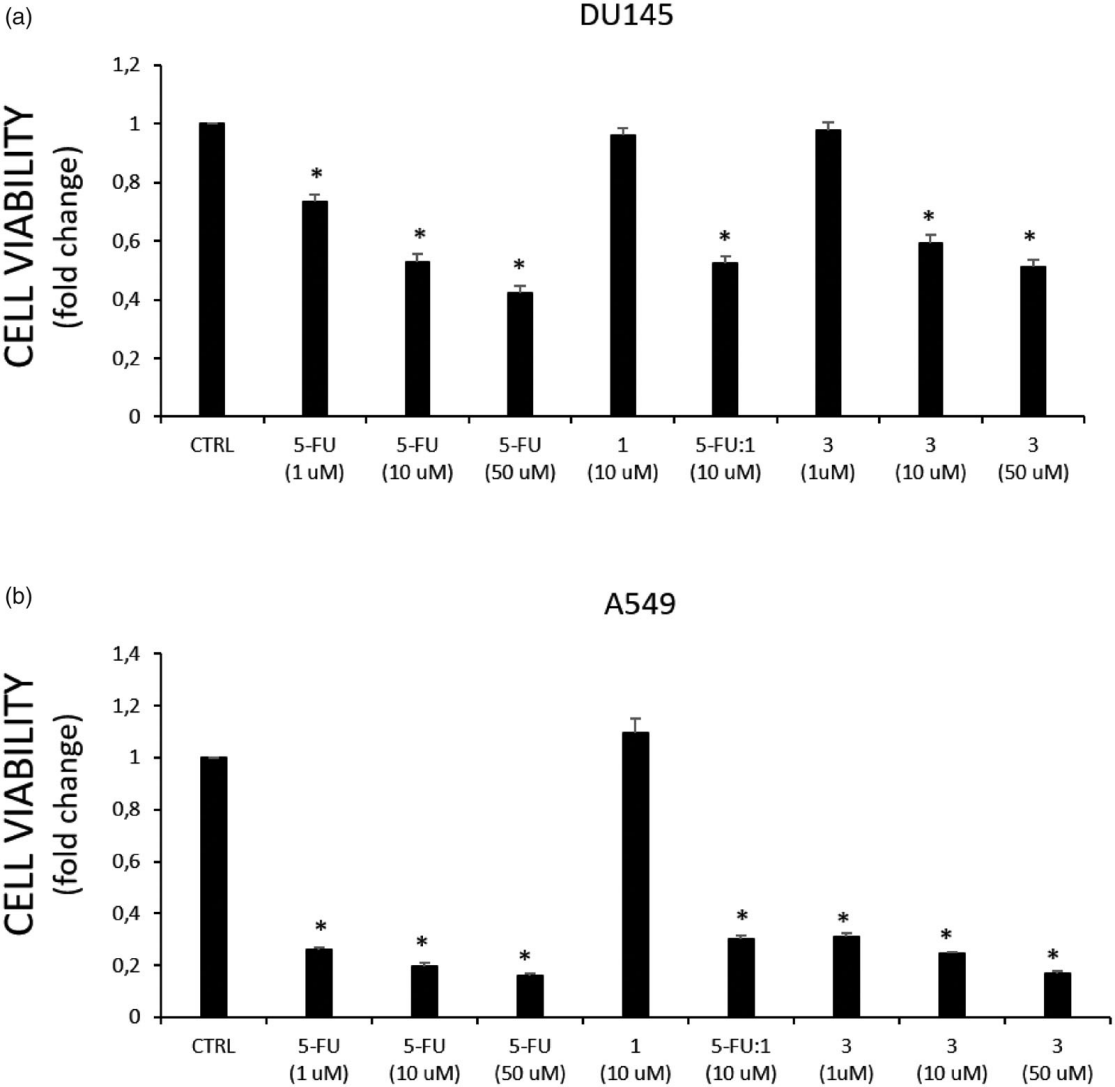
Effect on cell viability of tested compounds in (a) DU145, and (b) A549 cancer cells. Cell viability is expressed as fold change in viability from the control in treated cells (72 h). Data are presented as mean ± SEM (*n* = 8) of four independent experiments. * Significant *vs.* untreated control cells: *p* < 0.05.

Compound **3** was preliminarily assessed for its cytotoxic activity towards human prostate and lung cancer cell lines (DU145 and A549, respectively), in which the overexpression of the HO-1 has been confirmed^46^^,^^47^. Combination of parent compounds 5-FU and **1**, in a 1:1 ratio, was also evaluated and used for comparison. Briefly, cell lines were treated with the tested compounds at different concentrations (1, 10, and 50 μM). Cell survival was calculated compared to untreated controls for 72 h. At the end of treatment, cell viability was evaluated using the MTT assay. Results are depicted in Figure 4.

Compound **3** produced a significant dose-dependent effect on both cancer cell lines, similar to that of the reference drug 5-FU (Figure 4(a,b)). Similarly, the 5-FU:**1** combination showed a comparable effect on reducing cell viability of both 5-FU and **3** at the same dose, though no cytotoxic effect was exerted by the HO-1 inhibitor (**1**) towards the tested cancer cell lines (Figure 4(a,b)). The lack of biological activity on cell viability showed by the HO-1 inhibitor **1** is not an unexpected result since the HO-1 inhibition proved to be a valuable strategy not only to determine an intrinsic tissue-specific antiproliferative effect^48^, but also to potentiate the activity of existing chemotherapeutic drugs or to restore the sensitivity to anticancer agents in case of drug resistance^21–24^. On the other hand, the combination of 5-FU and HO-1 inhibitors in the same molecule might benefit patient compliance and therapy management.

**Figure 5.**
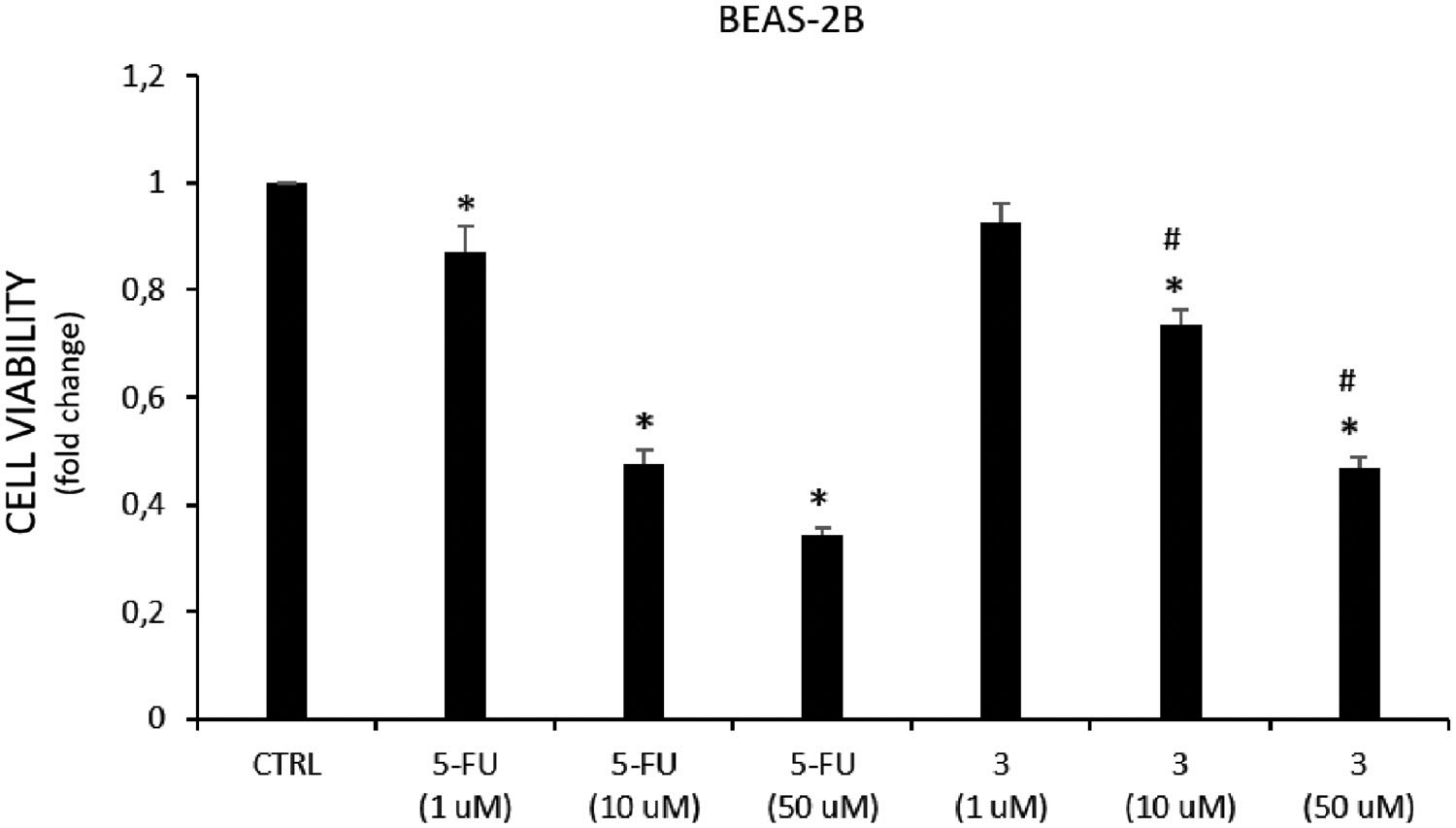
Effect on cell viability of tested compounds in BEAS cells. Cell viability is expressed as fold change in viability from the control in treated cells (72 h). Data are presented as mean ± SEM (*n* = 8) of four independent experiments. * Significant *vs.* untreated control cells: *p* < 0.05; ^#^ Significant *vs.* 5-FU treated cells: *p* < 0.05.

Remarkably, among the tested cancer cell lines, A549 cells resulted in more sensitivity to the treatment with 5-FU (IC_50_ = 0.98 ± 0.13 μM) and **3** (IC_50_ = 1.45 ± 1.04 μM) than DU145 cells (IC_50_ = 31.65 ± 0.92 and 46.93 ± 2.34 μM, respectively). Furthermore, to compare the cytotoxic effect against cancer *vs.* normal cell lines, compound **3** has been tested towards the non-tumorigenic human lung epithelial cell line (BEAS-2B) selected as a healthy cell model (Figure 5). Noteworthy, hybrid **3** showed a lower effect on BEAS-2B cells viability comparing to the 5-FU effects at the same doses. These results, together with the good *in vitro* and predicted pharmacokinetic properties of **3**, support our hypothesis that 5-FU/HO-1 hybrids might possess some advantages over 5-FU alone.

## Conclusions

Here, we described the synthesis of the first 5-FU/HO-1 hybrid (**3**) developed according to the mutual prodrug approach. Assessed molecular descriptors and predicted ADME/Tox properties for **3** suggested an overall drug-like profile. *In vitro* studies performed on **3** revealed that it was chemically stable in acid and neutral pH conditions, while it showed a suitable enzymatic hydrolysis rate in porcine esterase solution, with approximately 50% of **3** remaining after two hours (i.e. t_1/2_ = 136 min). Finally, **3** had comparable cytotoxicity to the parent drug 5-FU on both DU145 and A549 cancer cell lines, with a significantly improved selective toxicity against lung cancer cells (IC_50_ normal cell *vs*. IC_50_ cancer cells) compared to the reference drug 5-FU. To summarise, our data provided evidence to support the development of 5-FU/HO-1 mutual prodrugs as innovative potential anticancer agents. These findings warrant further studies in a broader panel of cancer cells, including 5-FU-resistant cells.

## Supplementary Material

Supplemental Material
