# Supplementary material for: Novel mutual prodrug of 5-fluorouracil and heme oxygenase-1 inhibitor (5-FU/HO-1 hybrid): design and preliminary in vitro evaluation
Source: J Enzyme Inhib Med Chem. 2021 Jun 24;36(1):1378–86. doi: 10.1080/14756366.2021.1928111 (PMC8231349; doi:10.1080/14756366.2021.1928111)
Supplement: Supplemental Material [file IENZ_A_1928111_SM4399.pdf]

Supplemental Material

Novel mutual prodrug of 5-fluorouracil and heme oxygenase 1 inhibitor (5-FU/HO-1 hybrid): design and preliminary *in vitro* evaluation

Loredana Salerno[a], Luca Vanella[a], Valeria Sorrenti[a], Valeria Consoli[a], Valeria Ciaffaglione[a], Antonino N. Fallica[a], Vittorio Canale[b], Paweł Zajdel[b], Rosario Pignatello[a], and Sebastiano Intagliata[a]*

[a]*Department of Drug and Health Sciences, University of Catania, Viale A. Doria 6, 95125 Catania, Italy;* [b]*Department of Organic Chemistry, Jagiellonian University Medical College, 9 Medyczna Str., 30-688, Kraków, Poland*

*corresponding author: s.intagliata@unict.it; Tel.: +39-095-738-4053

Table of contents

**Figures S1**, **S2:** ^{1}H NMR spectra of intermediate **2** and final compound **3**	S2
**Figures S3**, **S4:** MS spectra of intermediate **2** and final compound **3**	S3
**Figures S5-S7:** HPLC chromatograms of **1**–**3**	S4
**Table S1:** Percentage of compound remaining (**3**) at different pHs and time intervals	S5
**Figure S8:** Hydrolysis rate of **3** in PBS buffer (pH = 7.4)	S5
**Figures S9-S12:** representative HPLC chromatograms of the *in vitro* stability of **3** in PBS buffer (pH = 7.4) and porcine esterase solution	S5-S6
**Table S2:** Inhibitory potency of compounds **1**–**3** towards HO-1	S7

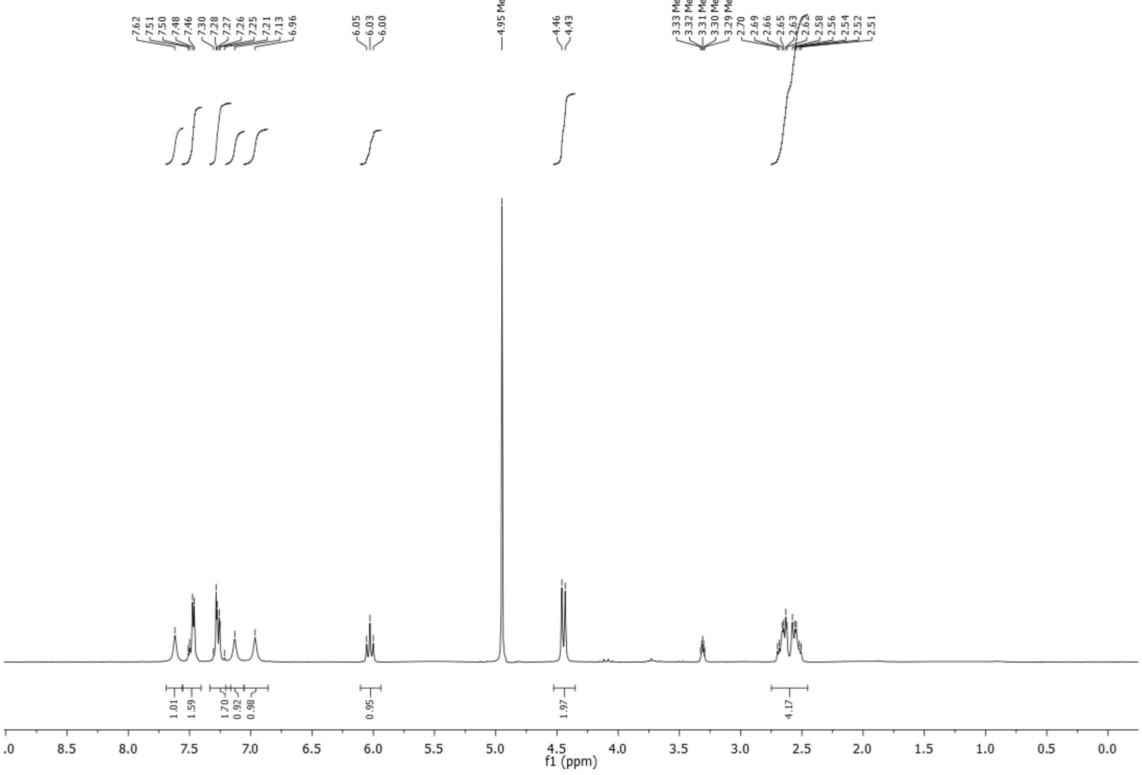

Figure S1. ^{1}H NMR spectrum of compound **2**

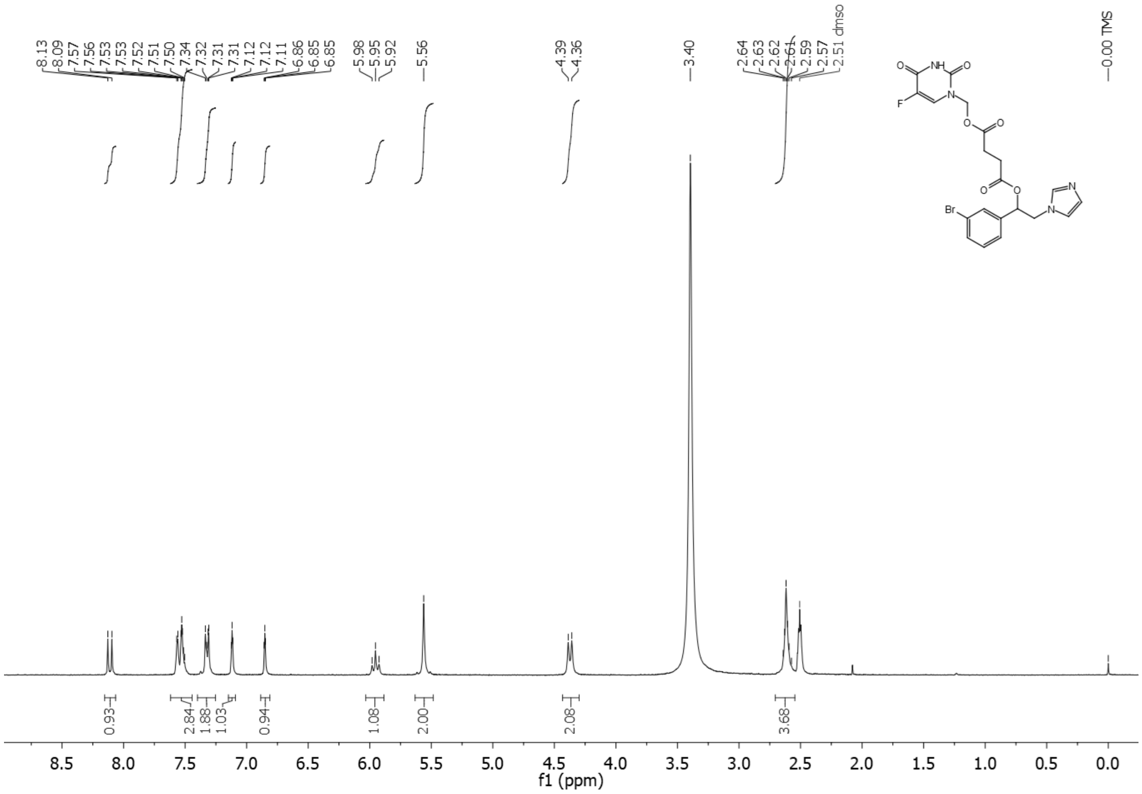

Figure S2. ^{1}H NMR spectrum of compound **3**

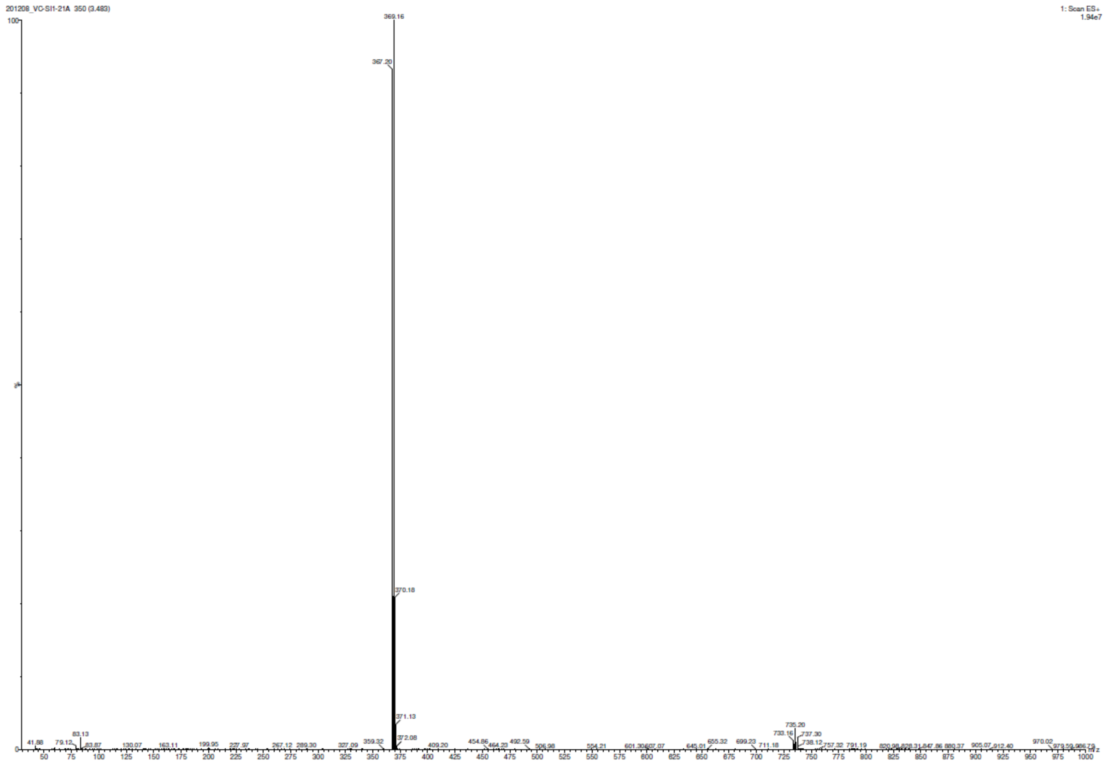

Figure S3. MS spectrum of compound **2**

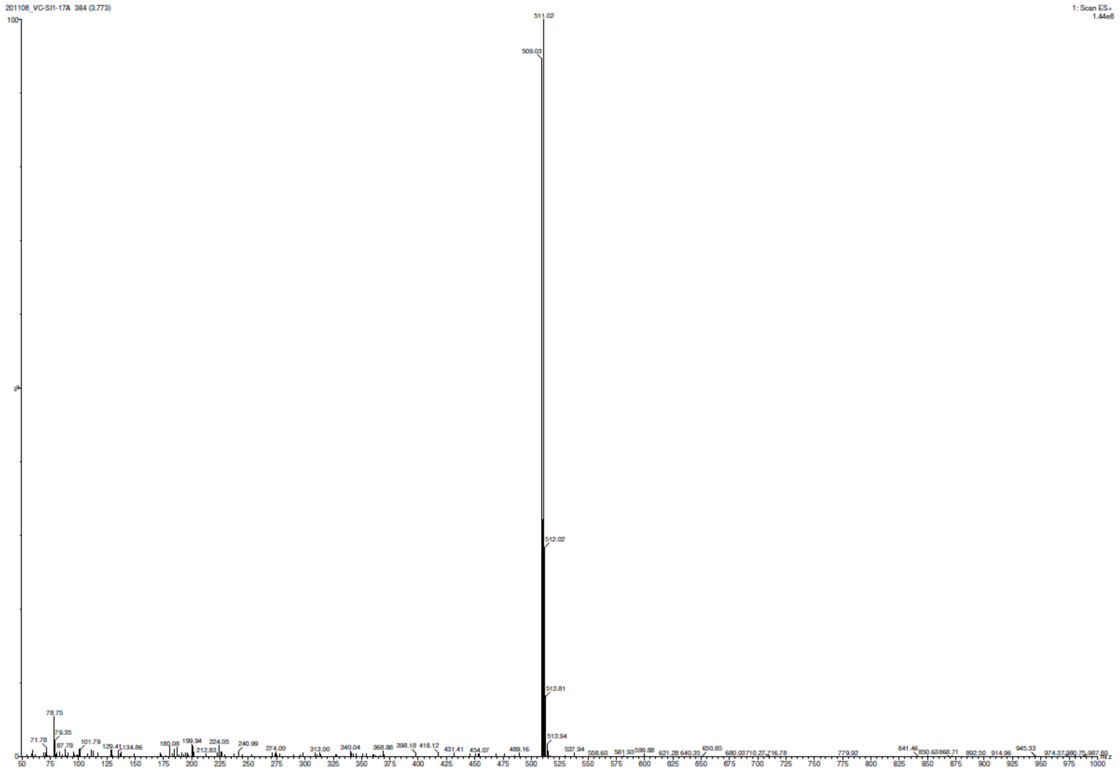

Figure S4. MS spectrum of compound **3**

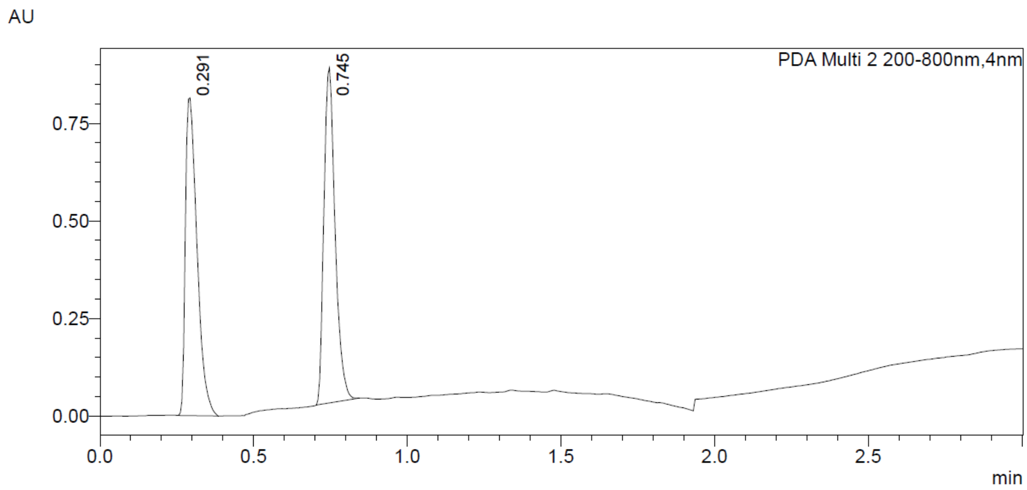

Figure S5. HPLC chromatogram of **1** (t_R = 0.745)

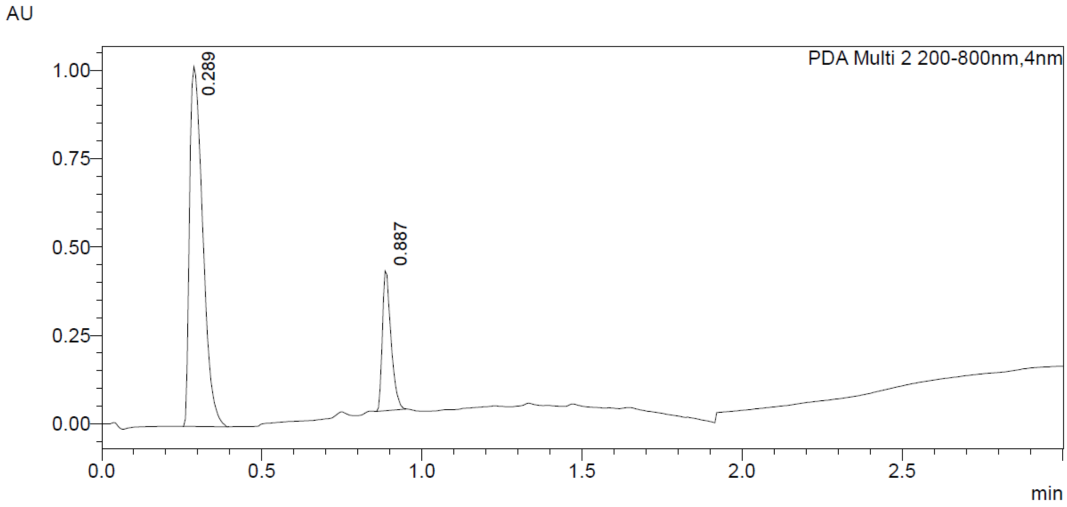

Figure S6. HPLC chromatogram of **2** (t_R = 0.887)

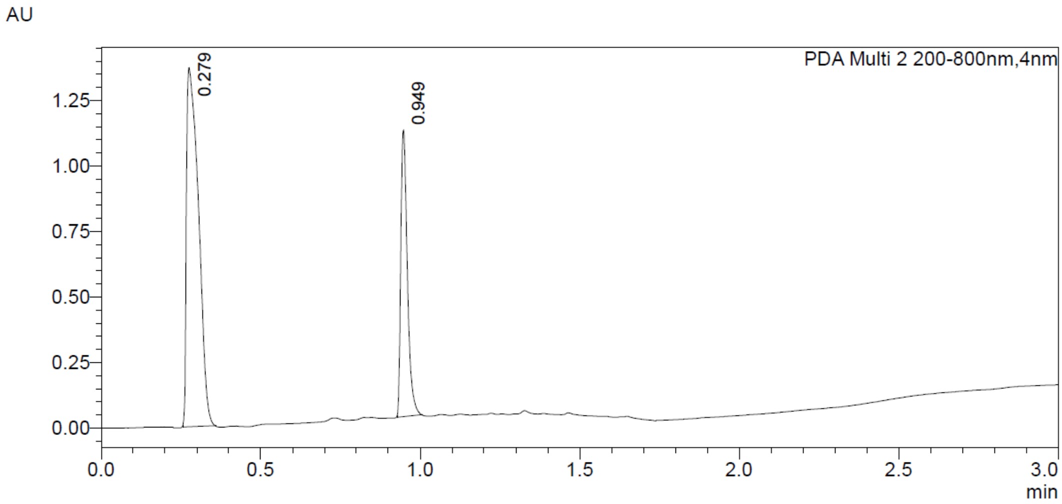

Figure S7. HPLC chromatogram of **3** (t_R = 0.949)

Table S1. Percentage of compound remaining (**3**) at different pHs over different time intervals.

**Time (h)**	**% Compound remaining** [1]		
	pH = 2.0	pH = 7.4	pH = 8.0
0	100 ± 0.0	100 ± 0.0	100 ± 0.0
24	94.2 ± 0.9	77.3 ± 3.9	50.4 ± 2.4
48	92.2 ± 0.6	71.2 ± 4.5	36.1 ± 2.1
72	86.8 ± 6.0	52.6 ± 3.8	19.7 ± 1.6
96	77.1 ± 6.5	34.7 ± 2.2	N.D.[2]
168	73.5 ± 4.7	18.0 ± 4.2	N.D.[2]

[1] Values represent the mean ± SEM of three independentxperiments;
[2] N.D. = not detected

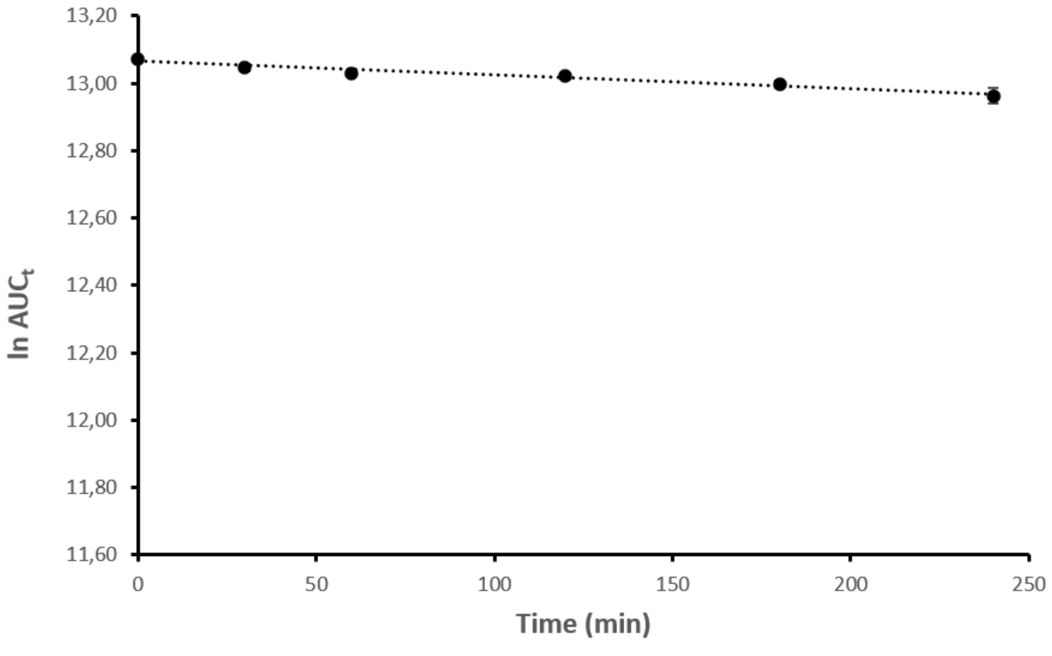

Figure S8. Hydrolysis rate of **3** in PBS buffer (pH = 7.4). A linear pseudo first-order plot of the ln AUCt *vs.* time was observed. $k = 0.41 \times 10^{-3}$ min^{-1}; $t_{1/2}$ = 1689 min; r = 0.979. Data are representative of three independent experiments and values are expressed in mean ± SEM.

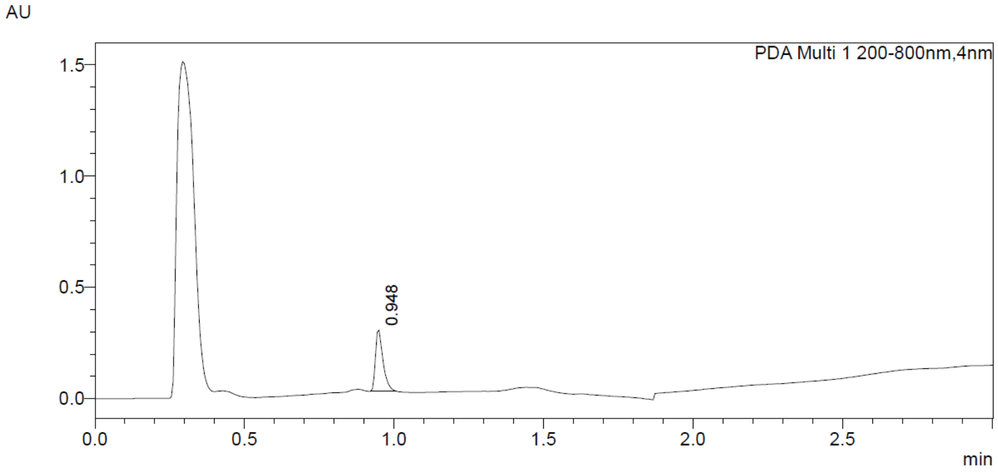

Figure S9. HPLC chromatogram of **3** (t_R = 0.948) immediately after its incubation in PBS buffer (pH = 7.4)

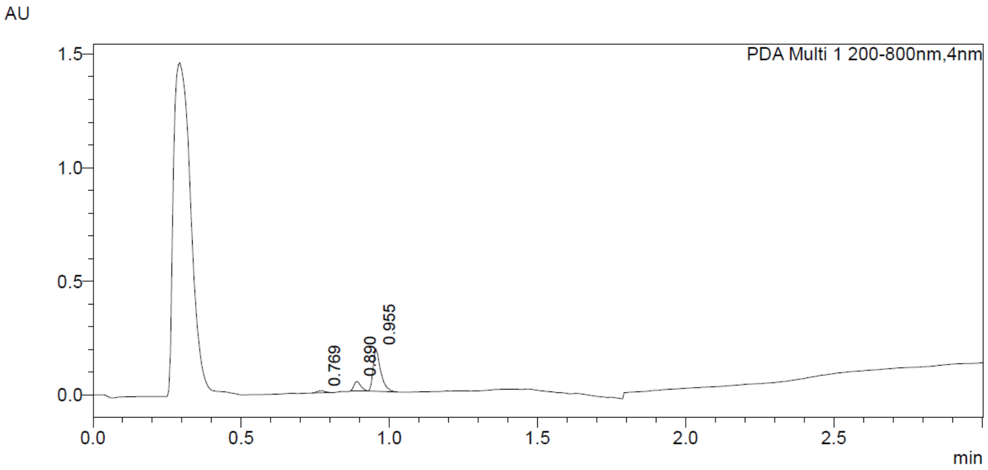

Figure S10. HPLC chromatogram of **3** (t_R = 0.955) 24 hours after its incubation in PBS buffer (pH = 7.4)

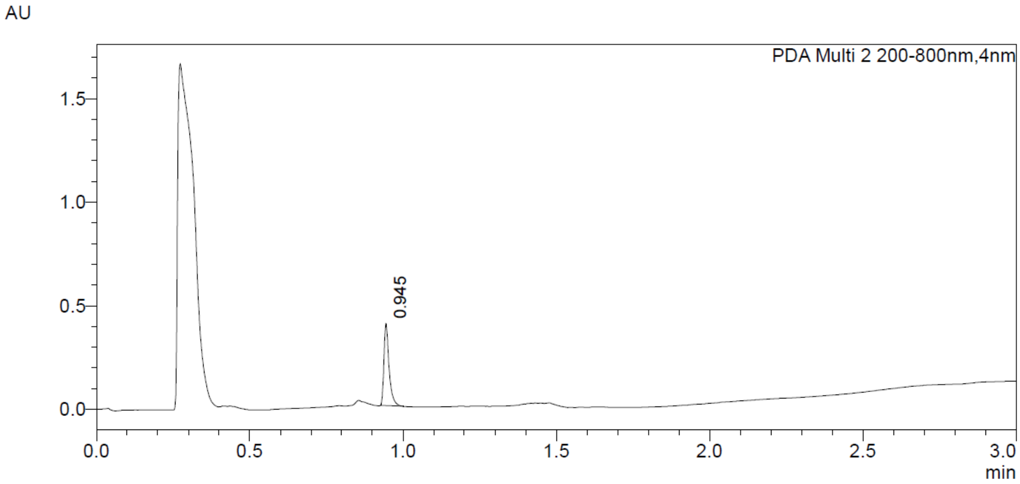

Figure S11. HPLC chromatogram of **3** (t_R = 0.945) immediately after its incubation in porcine esterase solution

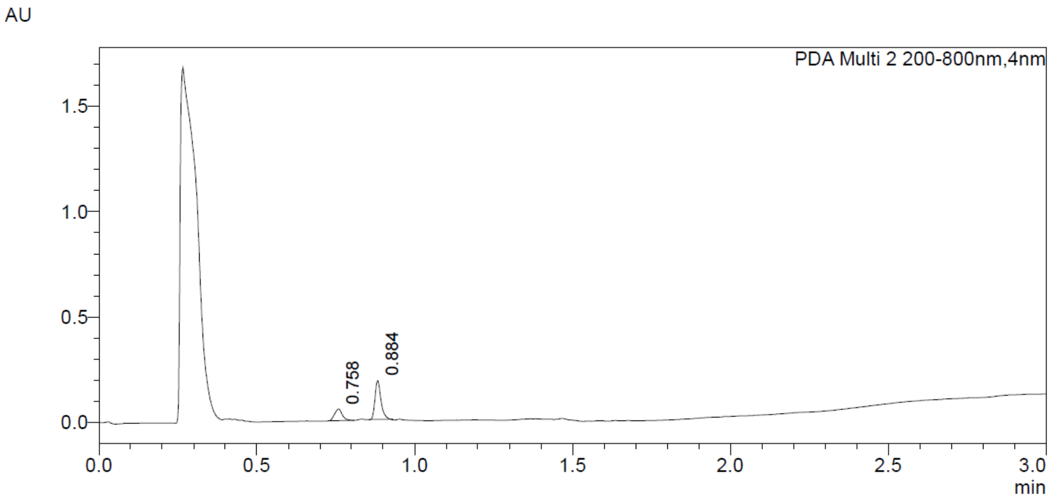

Figure S12. HPLC chromatogram of **3** (t_R = 0.952) 24 hours after its incubation in porcine esterase solution

Table S2. Inhibitory potency of compounds **1**–**3** towards HO-1.

Compound	HO-1 IC_{50} (µM) [1]
**1** [2]	0.4 ± 0.01
**2**	104.6 ± 5.8
**3**	82 ± 2.1

[1] Values represent the mean ± standard deviation (SD) of triplicate assays;
[2] Data from Greish *et al. Molecules* **2018**, *23*, 1209.
